# Novel application of stereotactic ablative radiotherapy using CyberKnife^®^ for early-stage renal cell carcinoma in patients with pre-existing chronic kidney disease: Initial clinical experiences

**DOI:** 10.3892/ol.2014.2129

**Published:** 2014-05-09

**Authors:** CHENG-HSIANG LO, WEN-YEN HUANG, HSING-LUNG CHAO, KUEN-TZE LIN, YEE-MIN JEN

**Affiliations:** Department of Radiation Oncology, Tri-Service General Hospital, National Defense Medical Center, Taipei 114, Taiwan, R.O.C.

**Keywords:** renal cell carcinoma, CyberKnife, renal function, chronic kidney disease, stereotactic ablative radiation therapy

## Abstract

The treatment of renal cell carcinoma (RCC) in patients diagnosed with chronic kidney disease (CKD) requires particular care in order to preserve the remaining renal function. The present study aimed to investigate the potential of a novel nephron-sparing treatment, which is capable of targeting tumors embedded deep within tissues. The present study analyzed three patients, with pre-existing CKD and multiple comorbidities, who were successfully treated for stage I RCC using the CyberKnife^®^ stereotactic ablative radiotherapy (SABR) system. The total prescribed dose was 40 Gy in five fractions administered over five consecutive days. Treatment efficiency was determined using computed tomography scans of the tumors and periodic measurements of the glomerular filtration rate over a period of 12–40 months. Local control, defined as a radiologically stable condition, was achieved in all patients. Lung metastasis was observed in one patient nine months after SABR; however, the side-effects were generally mild and self-limiting. One patient developed renal failure 26 months after SABR, while the severity of CKD was only marginally altered in the other two patients and renal failure did not occur. In conclusion, in the present study, SABR with CyberKnife^®^ was observed to be well tolerated in the patients, with an acceptable acute toxicity effect. Therefore, it may represent a potential therapeutic option for patients with early-stage RCC who have previously been diagnosed with CKD, but for whom other nephron-sparing treatments are contraindicated.

## Introduction

Renal cell carcinoma (RCC) is the most common form of kidney cancer and its incidence has risen markedly over the past decade (1). With recent advances in imaging technology, stage I RCC is currently acknowledged to account for ~60% of cases of RCC (2). In the early stage, tumors (size, <7 cm) are confined to the kidneys with no lymph node involvement, allowing for local treatment strategies.

According to recently published guidelines, partial nephrectomy is considered to be the standard treatment for clinical T1a and selected T1b tumors (3). Based on the importance of preserving the renal parenchyma and avoiding chronic kidney disease (CKD), patients with a single kidney, bilateral renal tumors or renal insufficiency are also candidates for partial nephrectomy, where technically possible (3,4). Percutaneous thermal ablation is an alternative technique to radical nephrectomy, however, its limitations with regard to exophytic tumors <4 cm in diameter and a complication rate of 6.9–13.5%, may preclude certain patients from this modality (5). Furthermore, these two nephron-sparing treatments are not suitable for certain patients with early-stage RCC, for example those with poor performance status or major comorbidities. A less or non-invasive modality is required for such patients as an alternative treatment.

RCC is considered to be a radioresistant malignancy and conventional radiotherapy has no curative role in the treatment of primary tumors. In addition to advances in radiotherapy techniques, stereotactic ablative radiotherapy (SABR), also termed stereotactic body radiotherapy, has yielded a favorable local control rate for primary and metastatic tumors in a variety of tissues, including radioresistant tumors, such as melanoma and RCC (6–16). The safety and efficiency of the local control of SABR in RCC has also been demonstrated in previous studies (6,11–15,17). However, few studies have investigated the effect of SABR on renal function and have been limited to patients with normal renal function (12). Furthermore, to the best of our knowledge, no study on SABR has focused on patients with RCC and pre-existing CKD, which represents a serious complication in the preservation of renal function. The current study investigated three patients with RCC and pre-existing CKD and presents the preliminary results of SABR using the CyberKnife^®^ image-guided radiosurgery system. The present study aimed to analyze the safety and feasibility of SABR using CyberKnife^®^ as well as its impact on renal function in patients with CKD.

## Patients and methods

### Patients

Three patients with CKD and stage I RCC were treated at the Stereotactic Radiosurgery System Center, Tri-Service General Hospital (Taipei, Taiwan) between August 2009 and February 2012. The patients included one male and two females, aged 68, 83 and 85 years, all with a Karnofsky index of ≥60 (18). All patients had moderate to severe CKD, with an estimated glomerular filtration rate (eGFR) <60 ml/min/1.73 m^2^, according to the Kidney/Dialysis Outcomes Quality Initiative (K/DOQI) classification (19). Patient 3 had undergone right radical nephrectomy for right renal pelvis urothelial carcinoma 22 years previously. All patients had been histologically diagnosed with clear cell RCC (CCRCC) using computed tomography (CT)-guided biopsy. An abdominal CT was performed prior to treatment in order to determine tumor size and staging. These patients had been refused radical surgery due to major comorbidities and pre-existing CKD. The demographic and staging data are presented in Table I. Patients provided written informed consent.

### Positioning and target delineation

Patients were placed in the supine position, immobilized using customized whole-body vacuum pillows (CIVCO Medical solutions, Kalona, IA, USA) and underwent planning CT with a 1-mm slice thickness. The gross tumor volume (GTV) and organs at risk, including the liver, bilateral kidneys (compartment involved excluded), stomach, small intestine, large intestine and spinal cord, were contoured using simulation CT. The GTV is defined as the radiographically visible tumor based on CT images and the clinical target volume (CTV) is the equivalent to the GTV. The planning target volume (PTV) was obtained by adding 1–3 mm to the corresponding CTV, with modification when dose-limiting organs overlapped (with the exception of the normal kidney).

### Treatment equipment and method

In all three cases, stage I CCRCC was treated using only the CyberKnife^®^ SABR system (Accuray Inc., Sunnyvale, CA, USA) with different tumor-tracking devices. Patient 1 underwent SABR with the aid of an abdominal compression device and vertebral tracking (X-sight; Accuray, Inc.) in order to minimize setup errors and diaphragmatic motion; therefore, limiting tumor movement during radiotherapy. Patients 2 and 3 were treated using the real-time respiration tracking technique (Synchrony; Accuray, Inc.). This technique involved the implantation of five fiducial markers in or near the tumor under CT guidance using a 19-G needle and local anesthesia, which acted as radiographic markers for the Synchrony tracking system. One week after implantation, planning CT scans were performed for these two patients. During the procedure, appropriate symptomatic treatments were administered to manage any complications, including nausea, fatigue or dizziness.

### Dose fractionation and dosimetric analysis

Treatments were administered in five fractions, with 8 Gy per fraction prescribed to the periphery of the PTV. Treatment planning was performed using the MultiPlan CyberKnife^®^ planning system version 2.1.0 (Accuray, Inc.). The dose constraint for the ipsilateral uninvolved kidney was less than a third of the volume of the unilateral normal kidney that received >15 Gy. Other organs, with dose limitations and their constraints, have previously been reported and are shown in Table II (9). With these dose-volume limitations, the PTVs were encompassed by the 72.0, 83.3 and 83.9% isodose curves. Dose-volume histograms provided the required data on dose distribution, and a conformity index (CI) and dose heterogeneity index (HI) were used for planning evaluation. The CI is defined as the ratio of the tissue volume that receives equal to or more than the prescription dose, to the tumor volume, which receives equal to or more than the prescription dose. The HI is defined as the ratio of the maximum dose to the prescription dose. The planning data are shown in Table III.

### Post-treatment follow-up

All patients were examined daily during treatment to assess acute toxicity effects. Subsequent to treatment, patients were followed up every 1–2 months for the initial 6 months and every 3–4 months thereafter. History taking, clinical examination and serum biochemistry analysis were performed at each follow-up. Toxicity was recorded based on the worst toxicity experienced and was graded according to the Radiation Therapy Oncology Group (RTOG) radiation injury grading criteria (19). Acute toxicity was defined as an adverse event occurring within three months of radiotherapy and late toxicity was defined as an adverse event occurring after three months (20). Surveillance CT scans were performed at 3–4-month intervals subsequent to SABR and the Response Evaluation Criteria in Solid Tumors (21) was used to assess the response.

### Renal function assessment

Renal function was assessed using the eGFR, which was calculated using the Modification of Diet in Renal Disease Study formula as follows: eGFR (ml/min/1.73 m^2^) = 186 × serum creatinine (S_cr_) - 1.154 × age - 0.203 × (0.742 if female) × (1.233 if Chinese) (22). The severity of CKD was graded according to the K/DOQI classification: Stage 1 (>90 ml/min/1.73 m^2^; kidney damage/normal GFR); stage 2 (60–89 ml/min/1.73 m^2^; kidney damage/mild decrease in GFR); stage 3 (30–59 ml/min/1.73 m^2^; kidney damage/moderate decrease in GFR); stage 4 (15–29 ml/min/1.73 m^2^; kidney damage/severe decrease in GFR); and stage 5 (<15 ml/min/1.73 m^2^; kidney failure) (20).

## Results

### Tumor response and survival

**Figure 1:**
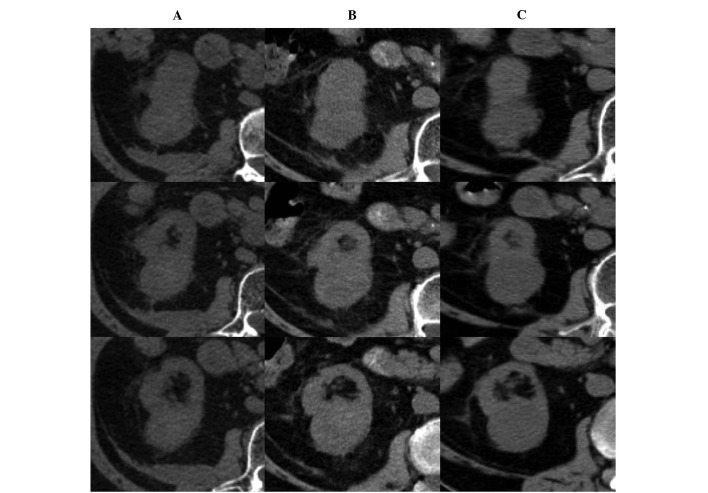
Computed tomography (CT) scan of patient 1 with renal cell carcinoma prior to and following stereotactic ablative radiotherapy (SABR). (A) Unenhanced scan prior to treatment shows a well-defined protruding mass in the right kidney, as well as a thin cortex and irregular contour of the kidney, which is consistent with chronic kidney disease. (B) CT scan 12 months and (C) 24 months following SABR. The patient was classified as stable following radiotherapy.

At the censor date, all patients were alive and their total follow-up times were 40, 13 and 12 months post-SABR. Non-enhanced abdominal CT scans were performed every three months to assess the tumor response, with results indicating that all patients had a stable condition in the area that was irradiated. Fig. 1 shows representative images of the patients pre- and post-SABR. Patient 3 developed asymptomatic multiple metastases nine months after SABR, for which sorafenib was administered for disease control.

### Toxicities

All acute toxicities were grade 1. Patient 3 had grade 1 nausea and dizziness following the administration of the first two fractions of SABR, however, these symptoms were self-limiting and rapidly improved following the completion of the course of radiotherapy. Patients 1 and 2 tolerated the treatment well and exhibited no adverse acute effects. After three months, toxicity analysis revealed no adverse late reactions. At the time of analysis, no grade 3 or 4 toxicity was observed.

### Renal function following SABR

No patients received dialysis up to the censor date. The eGFR pre- and post-SABR is shown in Fig. 1. In patient 1, at the 26-month follow-up the eGFR was found to have reduced from 17.51 to 12.28 ml/min/1.73 m^2^ [S_cr_, 3.4–4.6 mg/dl, (35%)] with the K/DOQI stage increasing from 4 to 5 (kidney failure) and the eGFR was predicted to reduce further. The other two patients showed little change in S_cr_ levels; however, the K/DOQI stage increased from 3 to 4.

## Discussion

Renal cancer is commonly associated with CKD as tumors impair renal function (23). Thus, patients with RCC are at a high risk of developing CKD complications, including cardiovascular diseases and renal failure, and mortality (24). For these reasons, it is of particular importance to preserve renal function while treating patients who have primary renal cancer with pre-existing CKD, without compromising their treatment response. The present study aimed to investigate a novel application for SABR using CyberKnife^®^ as a non-invasive, nephron-sparing treatment for stage I RCC in patients diagnosed with significant kidney dysfunction.

In the present study, the efficacy of SABR was retrospectively assessed in three patients diagnosed with stage I CCRCC (tumor size, 3.6–5.7 cm) and moderate to severe CKD. Local control was achieved in all three patients following the delivery of 40-Gy radiotherapy over five consecutive days. Distant metastasis was detected in one patient after nine months of follow-up. To the best of our knowledge, this is the first study to analyze the efficiency of SABR in patients with CKD and RCC, which is most commonly used for treating patients with RCC who have normal kidney function (17). The present study indicates that the same dosimetry may be used for patients with CKD through patient-specific optimization of the targeted area using the CyberKnife^®^ Multiplan system, which is a high precision radiation delivery system that spares the surrounding normal tissue. In patients with RCC with normal kidney function, the crude local control rate and estimated two-year local control rate following SABR CyberKnife^®^ treatment have been reported to be between 84 and 100%, and 86 and 100%, respectively (17). Overall, results from these studies are consistent with the treatment success reported in patients with RCC with normal kidney function (6,11–15,17).

The safety of SABR was assessed according to the RTOG radiation injury grading criteria for adverse effects and with regard to the preservation of renal function. The treatment was well tolerated in terms of general adverse effects, with only one patient reporting transient nausea and dizziness. The most important limiting factor of this treatment appears to be the initial level of renal function. While patient 1 had stage 4 CKD, the other two patients had stage 3. The patient with the most advanced CKD experienced a gradual loss of kidney function following treatment, which culminated in kidney failure after 26 months. The two patients with stage 3 CKD also exhibited altered S_cr_ levels following SABR, with the stage of CKD severity increasing from 3 to 4, but with no renal failure. The poor pre-SABR renal function in patient 1 may have contributed to this result and patients with impending renal failure may benefit from this type of treatment, which may delay the requirement for dialysis.

One important factor that should be considered in radiation therapy of renal tumors, regardless of the technique used, is the quantity of renal volume that should be spared to prevent the occurrence of renal failure. The QUANTEC group proposed that almost complete sparing of a substantial proportion of the kidney volume is associated with the preservation of renal function, even with the focal delivery of high-dose radiation, for example SABR, and a no dose constraint is recommended for kidney sparing during SABR (20). Preservation of function may be due to the compensatory increase in renal function of the spared kidney volume. Compensatory capacity is reduced with increases in the irradiated kidney volume (25). Although, to the best of our knowledge, no reports of clinically relevant symptomatic renal dysfunction following SABR have been reported to date (17), few studies have investigated kidney tolerance and the effects of SABR on renal function in patients with CKD.

The dose-volume constraints on the normal kidney during SABR for RCC are critical, however, have yet to be established. Cassady (26) proposed a threshold dose of 15 Gy for renal injury based on data on bilateral whole kidney irradiation. Although the safety of the administration of higher doses in partial kidney irradiation has been demonstrated, the majority of the data on partial kidney radiation tolerance is based on small fraction sizes (0.4–2.0 Gy per fraction) (20). Svedman *et al* (12) investigated kidney injury following SABR in seven patients, each with only one functioning kidney. With a maximum V15 of 37.3%, five patients were found to have stable renal function following SABR, whereas the other two exhibited modest changes in S_cr_ after two and six years of follow-up, without the requirement for dialysis or other medical intervention. The V15 in the unilateral normal kidney may be an appropriate dose-volume constraint in SABR treatment planning. Pre-existing renal insufficiency may further reduce kidney radiation tolerance to a variable degree, thus a more stringent dose-volume constraint may be required for patients with CKD compared with those used in previous studies (20). In the present study, in accordance with previous findings with SABR on HCC with certain modifications, the V15 at less than a third of the unilateral normal kidney was set as our dose constraint in SABR treatment planning, aiming to spare as much normal kidney volume as possible (9).

The limitations of the present study are its retrospective nature, low patient numbers and relatively short follow-up for renal function. It would be valuable to assess the impact of SABR on renal function in this patient population over a longer period of time and in a prospective manner.

In conclusion, the present study has demonstrated the preliminary findings for the local control, side-effects and renal function status following SABR using CyberKnife^®^ in patients with RCC and pre-existing CKD. Therefore, SABR may be an acceptable alternative treatment option in patients for whom nephron-sparing surgery is contraindicated.

## Figures and Tables

**Figure 2:**
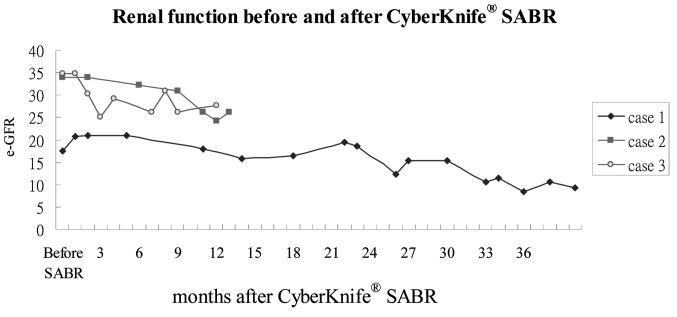
eGFR status in patients during follow-up. Values derived from the Modification of Diet in Renal Disease Study equation: e-GFR (ml/min/1.73 m^2^) = 186 × serum creatinine - 1.154 × age - 0.203 × (0.742 if female) × (1.233 if Chinese). eGFR, estimated glomerular filtration rate; SABR, stereotactic ablative radiotherapy.

**Table I:** Demographics and cancer staging in patients with renal cell carcinoma.

Case	Gender	Age	Tumor location	Tumor size (cm)^a^	Tumor stage	Comorbidities^b^	Pre-SABR eGFR (ml/min/1.73 m^2^)
1	Female	68	R’t lower pole	3.6	cT1aN0M0	Type 2 DM, HTN	17.51
2	Male	83	R’t lower pole	5.0	cT1bN0M0	Type 2 DM, HTN with CHF, L’t RAS after stent placement	33.88
3	Female	85	L’t lower pole	5.7	cT1bN0M0	R’t renal pelvis UCC after nephrectomy, Type 2 DM, HTN with CHF	34.79

a Tumor maximal diameter.

b Coexistent disease that may impair renal function.

SABR, stereotactic ablative radiotherapy; eGFR, estimated glomerular filtration rate; R’t, right; L’t, left; T, tumor; N, node; M, metastasis; DM, diabetes mellitus; HTN, hypertension; CHF, congestive heart failure; RAS, renal artery stenosis; UCC, urothelial carcinoma.

**Table II:** Dose-volume constraints for critical organs.

	Constraint		
	
Organ	Absorbed ratiation, Gy	Volume of organ receiving radiation	Maximum dose, Gy
Kidney	15	<1/3	-
Liver	<15	>700 cm^3^	-
Stomach	27	<5 cm^3^	<31
Small intestine	25	<5 cm^3^	<29
Large intestine	25	<5 cm^3^	<29
Spinal cord	-	-	<25

**Table III:** Dose-volume parameters for stereotactic ablative radiotherapy.

Case	CTV (cc)	PTV (cc)	Margin (mm)	Coverage (%)	V15 (%)	CI	HI	Total delivery time (h)
1	40.0	46.3	1	90.26	28.16	1.54	1.39	10.5
2	46.6	68.2	3	83.27	18.37	1.43	1.43	9.5
3	67.0	97.1	2	83.93	15.73	1.24	1.43	8.0

CTV, clinical target volume; PTV, planning target volume; V15, the percentage of ipsilateral normal kidney receiving >15 Gy; CI, conformality index; HI, homogeneity index.
